# Sandy Paradise or Rocky Hell? Investigating Soil Influence on *Thrinax radiata* Palm Groves from a Caribbean Island

**DOI:** 10.3390/plants14060853

**Published:** 2025-03-09

**Authors:** Gonzalo Castillo-Campos, M. Luisa Martínez, Jesús Pale Pale, José G. Garcia-Franco

**Affiliations:** 1 Red Biodiversidad y Sistemática, Instituto de Ecología A.C., Xalapa 91073, Mexico; gonzalo.castillo@inecol.mx (G.C.-C.); jose.pale@inecol.mx (J.P.P.); 2 Red Ecología Funcional, Instituto de Ecología A.C., Xalapa 91073, Mexico

**Keywords:** diversity, species richness, plant cover, plant height, coastal dunes, Mexico

## Abstract

Palm groves are abundant in southeastern Mexico and have significant economic and socio-cultural relevance. Studies on the production and management of palm groves are abundant in the area. However, an ecological approach is scarce but necessary for conserving these overexploited species. Palm groves are abundant on the island of Cozumel and develop on contrasting substrates: rocky and sandy ones. Thus, we aimed to explore how soil types affected community structure and composition. We sampled a total of 2900 m^2^ (29 plots of 10 × 10 m) of palm grove, 13 growing in sandy soil and 16 in rocky soil. We registered the species present in each plot, plant density, cover, height, and DBH. A hierarchical cluster analysis re-grouped the sampled plots based on their floristic affinities, and thus, we had 17 plots for the sandy substrate and 12 from the rocky substrate, which were used for later analyses. The dominant species in both palm groves was *Thrinax radiata*, and species richness was the same in both soil types (33 species). Contrasting substrates resulted in different community composition and structure. The palm groves from the sandy substrate had more vines but fewer shrubs and higher plant density, height, and DBH. The above suggests that the low availability of soil in the rocky substrate does not allow the same state of vegetation development that occurs in the sandy substrate.

## 1. Introduction

The Arecaceae family (palms), with around 2400 species, is widely distributed in tropical and subtropical regions [1,2,3]. In Mexico, around 95 species of palms have been recorded [4], 20 of which are distributed in the Yucatan Peninsula [5,6]. Given their significant economic and socio-cultural importance in this area, many studies focus on palm production, uses, and management [7,8,9,10,11,12,13]. However, information regarding the conservation status (and necessary restoration actions) of this group of plants is generally lacking, and in fact, several palm species are already in some endangered category due to over-extraction, disturbance, and habitat loss [14,15]. In this sense, ecological studies of this group of species are limited, and most of them aim at understanding the effect of disturbances in populations and knowing the factors that limit the distribution of some species [1,5,6,14]. There are no studies aimed at understanding the impact of environmental variables (such as type of substrate) on palm groves, which would be helpful for management and conservation programs.

In Mexico, most vegetation (floristic) studies that include palm species focus on the continental region of the Yucatan Peninsula, and very few include the vegetation of the Mexican islands. For example, some authors only mention the presence of *Thrinax radiata* (hereafter referred to as *Thrinax*) on the Island of Cozumel in the Mexican Caribbean [16,17,18,19,20,21]. In particular, the palm grove community of *Thrinax* has only been superficially studied in the interior part of the island [17]. However, its structure and species composition are largely unknown, especially for the palm groves growing on the coastal area in the eastern part of the island, where the palm is more frequent and abundant. The presence of these communities stabilizes the dunes and accumulates organic matter that promotes soil formation. Furthermore, their structure and dense cover reduce wind- and wave-induced erosion and help protect inland vegetation and human infrastructure [18].

Usually, palm grove communities are not very diverse, and one palm species is often dominant [6,22,23]. Several environmental characteristics determine the distribution and abundance of different palm species, such as environmental relative humidity, soil moisture, and soil type, among other factors [2]. The palm grove of Cozumel is distributed along the eastern coast in the protected natural area of the island and discontinuously south along the transversal road until the area known as Punta Sur [18,19]. The sandy substrate is dominant in this eastern coastal area, although gley soils are also present [24]. These soils have little organic matter and great permeability, but the depth of the sandy substrate is greater than the rocky gley soil in which outcrops of limestone sedimentary rock emerge [24].

Based on the above, the study goals of this study were (1) to describe the palm grove communities present on the eastern coast of the island of Cozumel and (2) to explore the role of different soil types in shaping community structure and composition of the *Thrinax* palm grove. We hypothesized that (a) palms would be dominant in the palm grove, independent of the soil type, and (b) because the rocky gley soil is shallower than the sandy substrate and thus limits plant growth, we expected a reduced species richness and lower palm cover and density in the rocky site than on the sandy one. To our knowledge, there are no studies exploring soil influence on palm groves of *Thrinax radiata*.

## 2. Results

### 2.1. Community Composition

**Figure 1:**
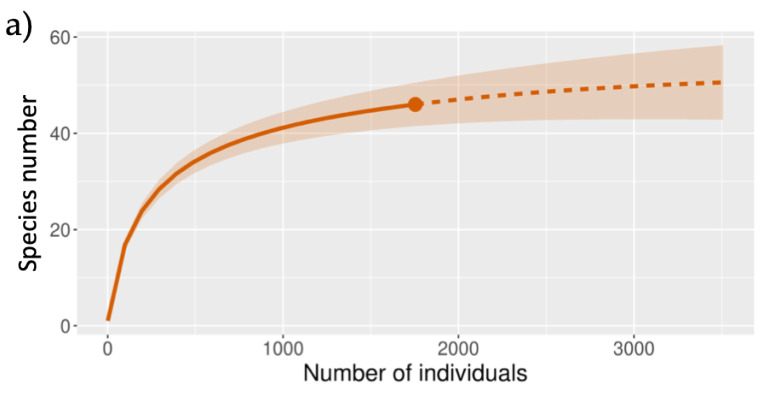

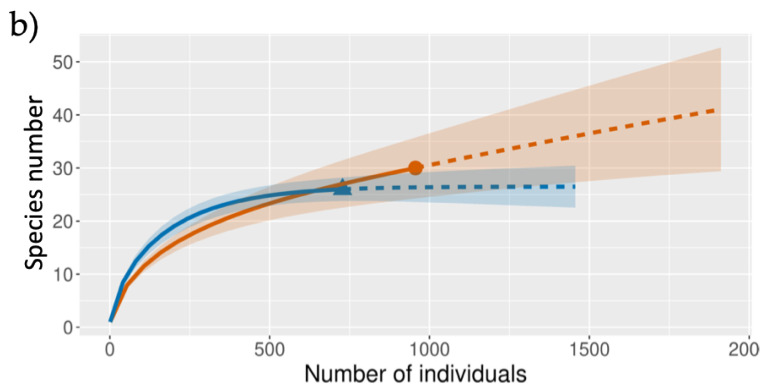
Rarefaction curve of a palm grove dominated by *Thrinax radiata* growing on the eastern coast of the island of Cozumel, Mexico. (**a**) Rarefaction curve of the total palm grove sampled. (**b**) Rarefaction curves for the sampled palm groves growing on two contrasting soil types (sandy–red and rocky–blue). The continuous line shows rarefaction and discontinuous line extrapolation. The shaded area indicates the 95% confidence interval.

We found 27 families, of which 46 species were woody, including 24 trees, 19 shrubs, and 3 vines, with a total surface of 2900 m^2^ (Table 1). The rarefaction curve indicates that the sampling effort was sufficient since the sample coverage was 0.995 (Figure 1a). The values of Chao 2 and Jackknife 1 suggest the potential presence of a maximum of 54 species for this plant community.

**Figure 2:**
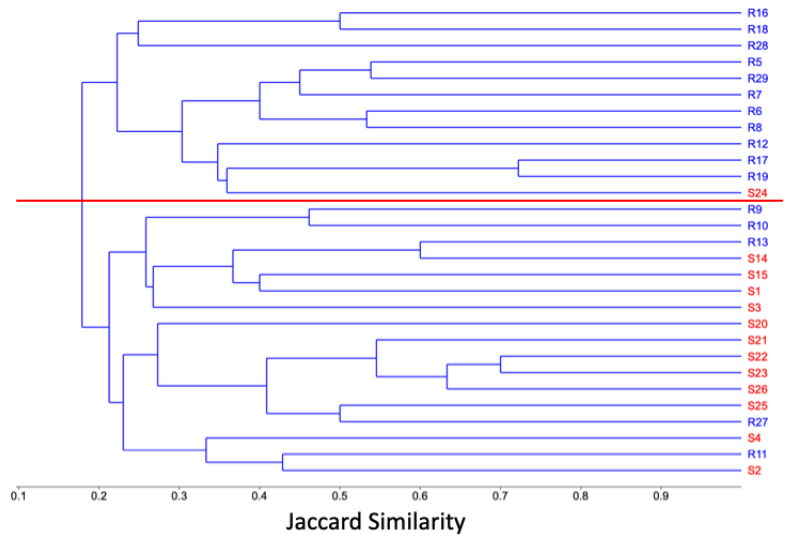
Hierarchical clustering analysis based on the Jaccard index. Two groups were formed based on the similarity in species composition between the plots sampled in different soil types. Letter S and red color correspond to the plots sampled on the sandy substrate. Letter R and blue color indicate the sampling plots from the rocky substrate. The red line divides the two groups at a similarity level of 0.19.

The hierarchical clustering analysis generated two groups that separated the sampling plots from each type of substrate according to their floristic composition (Figure 2). The first group comprised 17 sampling plots, almost all corresponding to the sandy substrate, while the second group comprised 12 sampling plots, almost all from the rocky substrate. These groups were used in the following analyses and comparisons. The rarefaction curves indicated that the sampling effort was more complete in the rocky substrate than in the sandy substrate (Figure 1b).

As expected, *Thrinax radiata* was the most abundant species in both substrates. The two palm groves differed in 33% of their species, most of which were represented by a small number of individuals (Table 1). The two floristic groups shared 20 species, representing 67% of the community. Some species, such as *Cordia sebestena*, *Gymnanthes lucida*, *Randia aculeata*, and *Sideroxylon americanum*, were more abundant in the sandy substrate, whereas *Neea psychotrioides* and *Pscidia piscipula* were more abundant in the rocky substrate. Hill numbers at 95% sampling efficiency indicated differences between palm groves established in the two substrates. The species richness (^0^*D*) and the abundant (^1^*D*) and dominant (^2^*D*) species numbers were higher in the rocky substrate than in the sandy substrate (^0^*D*_sandy_ = 15.2, ^0^*D*_rocky_ = 21.1; ^1^*D*_sandy_ = 2.5, ^1^*D*_rocky_ = 3.7; ^2^*D*_sandy_ = 1.5, ^2^*D*_rocky_ = 1.9).

### 2.2. Community Structure

Although the palm groves from both substrates had the same number of species (33 each), the community structure differed between them because the proportion of the different growth forms varied. The palm grove growing on the sandy substrate contained 18 species of trees, 12 shrubs, and 3 vines. In contrast, the palm grove from the rocky substrate had 18 species of trees, 15 shrubs, and no vines.

**Figure 3:**
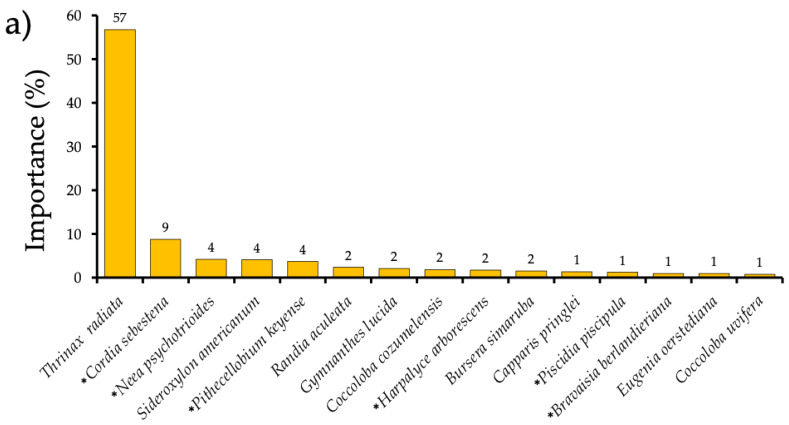

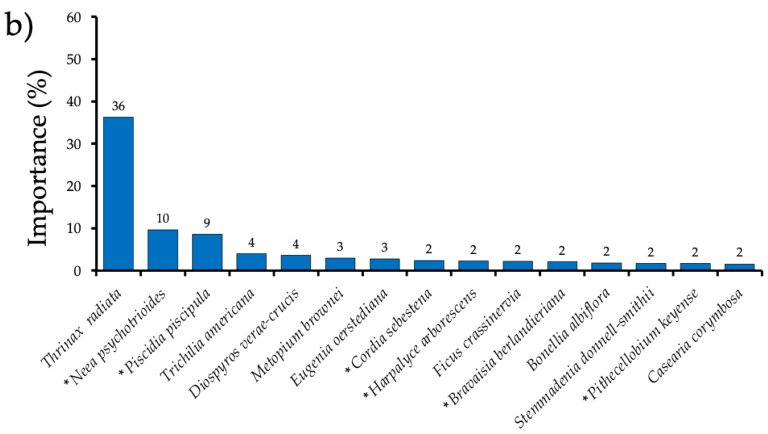
Importance value index of the species found in palm groves growing in sandy (**a**) and rocky (**b**) substrates on the island of Cozumel, Mexico. The numbers above the bars show the relative importance value per species. Asterisks indicate shared species between the two types of substrates. Group of soil type (sandy and rocky) according to the cluster analysis (see Section 4).

The importance value index (IVI) showed that *Thrinax radiata* was the most important species in the two substrates (Figure 3), thus defining the palmar community. However, the importance value of *Thrinax* on the sandy substrate was much higher than its value on the rocky substrate (57 and 36, respectively) (Figure 3). The subsequent species with higher IVI had notably lower values than those obtained for *Thrinax* and varied between the two substrates. After *Thrinax*, the most important species on the rocky substrate were *Neea psychotrioides*, *Piscidia piscipula*, *Trichilia americana*, and *Diospyros verae-crucis*, among other species. In contrast, after *Thrinax*, the most important species of the palm grove growing in the sandy substrate were *Cordia sebestena*, *Neea psychotrioides*, *Sideroxylon americanum*, *Pithecellobium keyense*, and *Randia aculeata*, among others. The species found in the two substrates maintained low importance values. However, some had higher values in the sandy substrate *(Cordia sebestena*) and some in the rocky substrate *(Neea psychotrioides, Piscidia piscipula*, and *Pithecellobium keyense*). One species, *Harpalyce arborescens*, had similar importance values in the two substrates (Figure 3).

**Figure 4:**
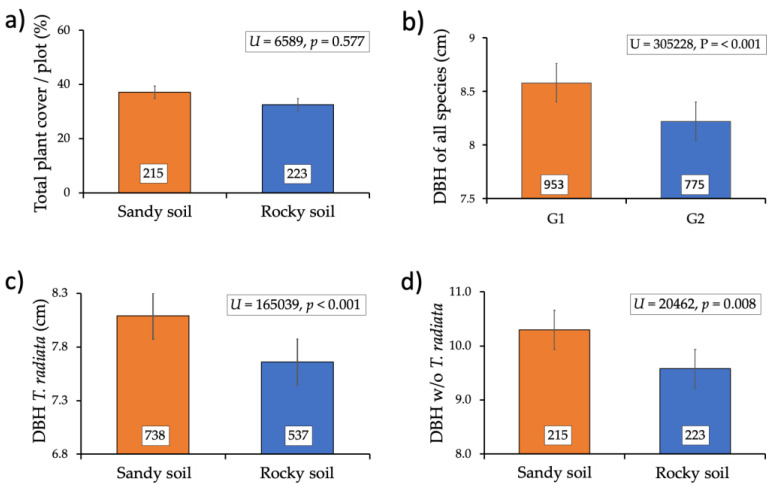
Palm grove attributes in two types of soil, sandy and rocky, on the island of Cozumel, Mexico. (**a**) Mean plant cover per plot; (**b**) mean DBH per plot for all plants found; (**c**) mean DBH per plot of *Thrinax radiata* palms; (**d**) mean DBH per plot for all plants found, excluding *Thrinax radiata*. The numbers in the boxes inside the bars indicate the number of plants in each case.

The density of plants, mean plant cover, and mean DBH per plot also varied between the substrates. We found 953 individuals on the sandy substrate and 775 on the rocky, yielding densities of 0.65 and 0.56 individuals /m^2^, respectively. Most of the individuals in both substrates were *Thrinax* palms (737 in the sandy substrate and 538 in the rocky substrate). Like the results for plant density, mean plant cover and DBH per plot were larger in the sandy than in the rocky substrates, although only differences in DBH were significant for all plant communities (Figure 4a,b). These trends in DBH held for the plant community without including *Thrinax* (Figure 4c) and for *Thrinax* alone (Figure 4d).

Finally, and contrary to the findings for plant density, plant cover, and DBH, there were no significant differences in the height of the palms (mean + EE; sandy 6.3 ± 0.3 m, rocky 6.5 ± 0.4 m; *t* = −0.578, *p* = 0.568) and the mean height of the entire community without the palm (sandy 5.4 ± 0.3 m, rocky 5.0 ± 0.2 m; *U* = 4849, *p* = 0.37).

## 3. Discussion

Our results corresponded to the two hypotheses. Despite the community’s high species richness, we found that palms are the dominant species in the palm grove of Cozumel Island. We also proved that species richness, plant cover, and plant density were reduced in the rocky soil substrate compared with the sandy soil one. The reason for the last one is that the rocky gley soil is shallower than the sand soil, thus limiting plant growth.

### 3.1. Community Composition and the Dominance of Thrinax radiata

The palm grove community located on the eastern coast of the Island of Cozumel has a high richness of plant species (46 woody species), with the dominance of the palm *Thrinax radiata*. This is the most important and physiognomically dominant species of the plant community (in terms of numerical dominance, coverage, height, and DBH). The rest of the species are not as abundant and do not represent relevant physiognomic elements.

Palms are abundant in the Yucatan Peninsula, where 20 species have been recorded. *Thrinax* is the palm with the amplest distribution in the peninsula and is conspicuous in the vegetation along with other palms [15,25]. However, in some places, a single species dominates the landscape [6]. Our results are consistent with the dominance of a single palm species observed in other palm groves [2]. However, it is striking that in the plant communities of the north and center of the eastern region of the Yucatan Peninsula, three palm species are dominant: *Thrinax radiata*, *Sabal yapa*, and *Chamaedorea seifrizii*, which are the three most abundant palms in the peninsula [5] growing on poorly developed soils like those existing on the island of Cozumel [5,6]. Thus, the absence of *Sabal yapa* and *Chamaedorea seifrizii* on the island may be associated with their limited dispersal by terrestrial vertebrates such as peccaries, squirrels, and mice [26,27,28].

Other environmental factors may be favoring the abundance of *Thrinax* and limiting the presence of other palms. For instance, tropical cyclones and hurricanes are regular meteorological phenomena in the Yucatan Peninsula; in the period of 2000–2012, 21 cyclones hit the coasts of the Yucatan Peninsula, 13 of them with a hurricane category [29], and they frequently affected Cozumel, especially along the island’s eastern coast [18]. Besides human infrastructure, hurricanes also have long-lasting effects on plant communities: they induce mortality and open gaps in the canopy and thus alter composition and structure [30,31,32,33,34,35]. Previous observations have shown that palms are tolerant to the impact of hurricanes, especially *Thrinax radiata*, which had a high survival rate after Hurricane Wilma in 2005 [36,37]. Thus, it is likely that the dominance of *Thrinax* in the palm grove of Cozumel is also associated with this species’ ability to better tolerate the limiting environmental conditions of the island compared with the other palms and associated species [2]. The result is a *Thrinax*-dominated palm grove with high species richness but a reduced abundance.

### 3.2. Community Structure

Our results showed that the palm groves from the eastern coast of Cozumel are very well defined by the two types of substrates (sandy and rocky) that are dominant in the area [18,22]. In general, these soils are poor in nutrients and have high infiltration, but even so, the accumulation of organic matter and sand, as well as the exposure of limestone rock, varies over relatively short distances. These factors are considered determinants in the richness and structure of plant communities [38,39] and, consequently, modify the richness of palm species in Cozumel and other continental nearby sites in the Yucatan Peninsula [6].

Although *Thrinax radiata* is the dominant physiognomic element in the two substrates and the two palm groves have the same number of species (33), there are some differences in their abundance and dominance and in the composition and structure of the two communities (Table 2). Our results showed that shrubs are more abundant on the rocky substrate than the sandy substrate, but vines are only present in the palm grove that grows on the sandy substrate. Furthermore, plant and palm density is higher in the sandy substrate, where the tallest and thickest individuals are present, while in the rocky substrate, the plant density is lower, and plants are thinner and shorter. The low availability of soil in the rocky substrate does not allow the same vegetation development that occurs in the sandy substrate. Coastal sandy substrates can be enriched with the accumulation of leaf litter from the vegetation cover and their decomposition by crabs and microorganisms [40,41], and although nutrient availability could take some time [42], a similar process may be occurring in our study area, producing the structural and compositional differences between the palm groves growing in sandy and rocky soils in Cozumel.

In particular, the reduction in the size and importance value of *Thrinax radiata* and the associated species growing in the rocky substrate draws our attention. Further studies are required to include the physical and chemical analyses of the soil, combined with the plant’s responses to these differences in soil characteristics. On the other hand, it is necessary to carry out focal studies to explore the unique distribution of the species that only occurred in one of the substrates (e.g., *Trichilia americana* in the rocky substrate and *Sideroxylon americanun* in the sandy substrate) to identify the factors that limit their presence in both substrates.

### 3.3. Caveats of the Study

This study shows how palm groves growing on rocky vs. sandy soils differ in number of shrubs, vines, plant cover, plant density, plant height, and DBH (Table 2). However, the differences between soil types and palm groves were not clearcut. Some plots from the sandy soil were more like those from the rocky plot and vice versa. These trends are probably the result of species exchange between plots and a gradient in soil attributes. A detailed soil analysis would further help explain the differences between palm groves growing on predominantly rocky and sandy soils. For instance, besides the relative rockiness of the surface, community differences could be associated with, for example, nutrient content and depth of aerobic and anaerobic soil. In addition to soil analysis, the rates of propagule flow between plots would also provide information on the dynamics of these palm groves on a meta-community scale.

Despite these limitations, our results help distinguish how palm grove communities are heterogeneous even though one species is dominant. Such variability is most likely associated with differences in soil attributes. Experimental data would help demonstrate this correlation and find its causes.

## 4. Materials and Methods

### 4.1. Study Site

**Figure 5:**
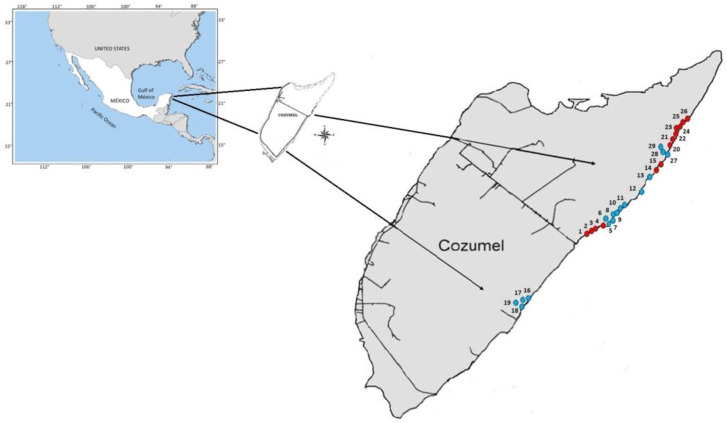
Location of the island of Cozumel in the Mexican Caribbean. Red dots show sampling plots with sandy substrate, and blue dots show sampling plots with rocky substrate. The numbers correspond to the sampling plots.

The island of Cozumel is the third largest island in Mexico, with around 480 km^2^. It is in the Mexican Caribbean, 17.5 km from the coast of the Yucatan Peninsula in the Mexican state of Quintana Roo (20 16′–20 36′ N, 86 44′–87 02′ W; Figure 5), and has always been isolated from the mainland [43]. The climate on the island is warm and humid with rain in summer; tropical storms and cyclones occur with some frequency during the summer [44]. The average annual temperature is 25.5 °C, with slight seasonal variation, and the average annual precipitation is 1570 mm [45,46]. Cozumel is an island of coral origin made up of sediments and calcareous rocks of marine origin from the recent Tertiary and Quaternary [19]. These characteristics have allowed the development of various types of vegetation with high biodiversity and many endemisms. The main vegetation types are medium-sized sub-evergreen rainforests, mangroves, coastal dune vegetation, and palm groves [16,19,20,21,47,48,49]. The study occurred on the island’s eastern coast, where *Thrinax radiata* populations are established in predominantly sandy and rocky sedimentary rock substrates (Figure 5).

### 4.2. Methods and Field Work

**Figure 6:**
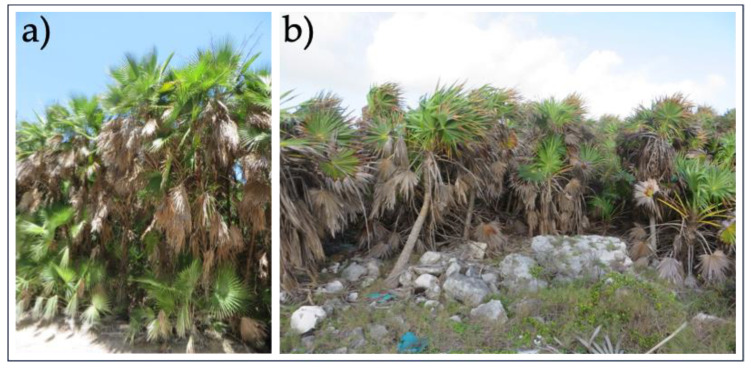
View of the palm grove community of *Thrinax radiata* on the island of Cozumel, Mexico. (**a**) Sandy soil, (**b**) rocky soil.

We used 10 × 10 m (100 m^2^) quadrats for vegetation sampling. The quadrats were distributed along the east coast of Cozumel (Figure 5), where the largest populations of *Thrinax radiata* occur. We established 13 plots on the sandy substrate (total sampled area, 1300 m^2^) and 16 on the rocky (total sampled area, 1600 m^2^) (Figure 6). The total palm grove surface sampled added 2900 m^2^, corresponding to 0.29 hectares. The plots were separated from each other by at least 25 m and distributed along the palm grove growing in the two substrates. We considered it sandy if the plot’s surface was covered in more than 60% of exposed sand; in contrast, it was considered rocky if more than 60% of the surface was observed with exposed rock. Subsequently, we recorded all woody species present and the height of the tallest individual for each species. Afterward, the total number of individuals of each species present was quantified. We measured the diameter at breast height (DBH) at a height above ground of 1.30 m in each plant with a trunk diameter greater than or equal to 2.5 cm. Finally, the total cover of each species was estimated as the sum of the crown projection of all individuals in the area within the sampling plot.

Because the island of Cozumel is a Biosphere Reserve and many of the sampling plots were in protected areas (Figure 5) [18,50], collecting specimens to identify and support the determinations was impossible. Therefore, most of the recorded species were identified “in situ” during sampling based on the botanical knowledge of one of the participants (GCC). When a species was not recognized, we used photographs of the complete plant and/or its reproductive structures for identification and consulting published floristic lists of Cozumel and the Yucatan Peninsula [38,48,51,52,53] and its comparison with herbarium specimens available in electronic databases (MEXU, XAL).

### 4.3. Data Analyses

First, we performed a rarefaction–extrapolation analysis using the iNEXT program available online [54]. In this analysis, we included all the species recorded in the two substrates since we wanted to know the completeness of the sampling and the coverage of the sampling carried out in the palm grove. Subsequently, a hierarchical clustering analysis (UPGMA) based on the Jaccard similarity index (presence–absence) was carried out to explore whether the plots sampled on the same type of substrate were grouped according to the similarity of their species composition. As this analysis mostly grouped the plots according to the substrate where they were located, we decided to carry out the following analyses and comparisons based on these two floristic groups differentiated by the type of substrate (sandy and rocky). These analyses were performed with PAST 4.11 [55]. Later, we conducted a rarefaction–extrapolation analysis for each floristic group formed by the cluster analysis.

We calculated the species diversity for each palm grove group based on Hill’s numbers of order *q* [56]. The three components of the effective number of species were obtained: ^0^*D* = observed richness, ^1^*D* = common or abundant species (equivalent to exponential of the Shannon Diversity index), and ^2^*D* = dominant species (equivalent to the inverse of Simpson’s Diversity Index). This analysis was conducted using the data obtained from the rarefaction (interpolation) and extrapolation (prediction) procedures for each palm grove group using iNEXT [54].

We calculated the importance value index (IVI) for each species found in each soil type to identify the species that contribute most to the differentiation of the two groups by applying the following equation:

IVI*_i_* = (*RD_i_* + *Rf_i_* + *RC_i_*)/3, (1)

where *RDi* is the relative density of species *i*, *Rfi* is the relative frequency of species *i*, and *RCi* is the relative cover of species *i*. The value obtained from the sum was divided by three to obtain percent importance values, ranging from 1 to 100. Subsequently, we only plotted the species with the highest IV values.

Finally, mean plant height, DBH, and plant cover measured in the rocky and sandy palm groves were compared through *t*-test and Mann–Whitney a posteriori contrasts when the data did not meet the normality and/or homogeneity assumptions. The analyses were carried out with the Jamovi 2.3 program [57].

## 5. Conclusions

Although *Thrinax radiata* is a species of economic and cultural importance among the Mayans and is used for construction in the Yucatan Peninsula, the palm grove of Cozumel is in a good state of conservation and reaches high densities along the eastern coast of the island. The good conservation status is due to several factors. Its location in the conservation area of the island and its designation as a threatened species by the official Mexican standard [14] have protected *Thrinax* from the inhabitants’ use and over-exploitation.

Our results confirm that contrasting substrates resulted in different community compositions and structures of two palm groves, even though *Thrinax* was dominant in both. The palm groves from the sandy substrate had more vines but fewer shrubs and higher plant density, height, and DBH. The above suggests that the low availability of soil in the rocky substrate does not allow the same state of vegetation development that occurs in the sandy substrate.

## Figures and Tables

**Table 1:** Families and plant species registered in a palm grove on the island of Cozumel, Mexico. Growth form and number of individuals found in each soil type group (sandy and rocky) are shown after the cluster analysis (see Section 4). The largest values are highlighted in bold and with an asterisk.

Family	Species	Growth Form	Sandy	Rocky
Acanthaceae	*Bravaisia berlandieriana* (Nees) T.F. Daniel	Shrub	3	5
Anacardiaceae	*Metopium brownei* (Jacq.) Urb.	Tree	1	7
Apocynaceae	*Echites yucatanensis* Millsp. ex Standl.	Vine	1	0
	*Stemmadenia donnell-smithii* (Rose) Woodson	Tree	0	5
Arecaceae	*Sabal yapa* C. Wright ex Becc.	Tree	1	0
	*Thrinax radiata* Lodd. ex Schult. & Schult. f.	Tree	737 *	538 *
Aristolochiaceae	*Aristolochia maxima* Jacq.	Vine	1	0
Asteraceae	*Eupatorium albicaule* Sch. Bip. ex Klatt	Tree	1	3
Burseraceae	*Bursera simaruba* (L.) Sarg.	Tree	4	4
Capparaceae	*Capparis flexuosa* (L.) L.	Shrub	0	4
	*Capparis indica* (L.) Druce	Shrub	1	3
	*Capparis pringlei* Briq.	Shrub	3	2
Combretaceae	*Conocarpus erectus* L.	Tree	3	0
Cordiaceae	*Cordia sebestena* L.	Shrub	68 *	5
Ebenaceae	*Diospyros verae-crucis* (Standl.) Standl.	Tree	0	15 *
Erythroxylaceae	*Erythroxylum bequaertii* Standl.	Tree	3	4
Euphorbiaceae	*Gymnanthes lucida* Sw.	Tree	14	2
Fabaceae	*Dalbergia brownei* (Jacq.) Schinz	Shrub	0	1
	*Erythrina standleyana* Krukoff	Tree	2	0
	*Harpalyce arborescens* A. Gray	Shrub	5	7
	*Piscidia piscipula* (L.) Sarg.	Tree	6	38 *
	*Pithecellobium keyense* Britton	Tree	**6**	3
Malpighiaceae	*Malpighia emarginata* DC.	Shrub	1	0
Malvaceae	*Hibiscus rosa-sinensis* L.	Shrub	0	2
Meliaceae	*Trichilia americana* (Sessé & Moc.) T.D. Penn.	Tree	0	21 *
Moraceae	*Ficus americana* Aubl.	Tree	0	7
	*Ficus crassinervia* Desf. ex Willd.	Tree	0	14
	*Ficus obtusifolia* Kunth	Tree	0	4
Myrtaceae	*Eugenia axillaris* (Sw.) Willd.	Tree	3	0
	*Eugenia oerstediana* O. Berg	Tree	7	11
Nyctaginaceae	*Neea psychotrioides* Donn. Sm.	Shrub	17	40 *
	*Pisonia aculeata* L.	Tree	2	0
Passifloraceae	*Passiflora biflora* Lam.	Vine	1	0
Polygonaceae	*Coccoloba cozumelensis* Hemsl.	Tree	9	3
	*Coccoloba diversifolia* Jacq.	Tree	0	2
	*Coccoloba uvifera* (L.) L.	Tree	1	0
Primulaceae	*Bonellia albiflora* (Lundell) B. Ståhl & Källersjö	Shrub	2	7
	*Parathesis cubana* (A. DC.) Molinet & M. Gómez	Shrub	0	1
Rubiaceae	*Chiococca alba* (L.) Hitchc.	Shrub	0	3
	*Randia aculeata* L.	Shrub	13	4
Salicaceae	*Casearia corymbosa* Kunth	Shrub	0	5
	*Casearia yucatanensis* (Standl.) T. Samar. y M.H. Alford	Shrub	1	0
Sapotaceae	*Manilkara zapota* (L.) P. Royen	Tree	1	0
	*Pouteria campechiana* (Kunth) Baehni	Tree	1	3
	*Sideroxylon americanum* (Mill.) T.D. Penn.	Shrub	31 *	2
Verbenaceae	*Lantana involucrata* L.	Shrub	3	0

**Table 2:** Summary of the differences in community composition and structure of two *Thrinax radiata* palm groves growing on contrasting substrates (sandy and rocky) on the island of Cozumel, Mexico. Values are mean ± EE. Letters in bold and asterisk indicate significantly higher values. U Mann–Whitney *p* < 0.05.

Community Attributes	Sandy	Rocky
Species richness	33	33
Trees (no. of species)	18	18
Shrubs (no. of species)	12	15 *
Vines (no. of species)	3 *	0
IVI *Thrinax*	57 *	36
Total number of individuals	953 *	775
Density (individuals/m^2^)	0.65	0.56
Number of *Thrinax* palms	738 *	538
Total plant cover (%)	37.2 * ± 3.6	32.6 ± 2.7
DBH all species (cm)	8.6 * ± 0.2	8.2 ± 0.3
DBH all species without *Thrinax* (cm)	10.2 * ± 0.6	9.5 ± 1.0
DBH *Thrinax* (cm)	8.1 * ± 0.1	7.7 ± 0.1
Community Height (m)	5.55 * ± 0.2	5.16 ± 0.2
Palm height (m)	6.3 ± 0.3	6.5 * ± 0.4
Community height without *Thrinax* (m)	5.4 * ± 0.3	5.0 ± 0.2

## Data Availability

The data used in this article are available upon request to the authors.
